# Psychological Sciences section of The Journals of Gerontology Series B

**DOI:** 10.1093/geroni/igaf122.1234

**Published:** 2025-12-31

**Authors:** Duke Han

**Affiliations:** University of Southern California, Los Angeles, California, United States

## Abstract

The Psychological Sciences section of The Journals of Gerontology, Series B publishes articles on development in adulthood and old age that advance the psychological science of aging processes and outcomes. Articles in the journal have clear implications for theoretical or methodological innovation in the psychology of aging or contribute significantly to the empirical understanding of psychological processes and aging. Areas of interest include, but are not limited to, attitudes, clinical applications, cognition, education, emotion, health, human factors, interpersonal relations, neuropsychology, neuroscience, perception, personality, physiological psychology, social psychology, and sensation. Applied research with theoretical significance is welcome, as are conceptually interesting examples of cutting-edge analytic approaches. Manuscripts reporting work that relates behavioral aging to neighboring disciplines are also appropriate. The Journal publishes three types of articles: (a) Research Articles, reportings on original research, preferably with multiple studies and/or samples, (b) Research Reports, for brief presentations generally of single studies, and (c) New Directions in Aging Research—reviews of cutting-edge topics with theoretical or methodological implications. See word and page limitations below. All submissions are peer-reviewed, with final decisions made by the Editor.

